# Human Variants in the Neuronal Basic Helix-Loop-Helix/Per-Arnt-Sim (bHLH/PAS) Transcription Factor Complex NPAS4/ARNT2 Disrupt Function

**DOI:** 10.1371/journal.pone.0085768

**Published:** 2014-01-17

**Authors:** David C. Bersten, John B. Bruning, Daniel J. Peet, Murray L. Whitelaw

**Affiliations:** School of Molecular and Biomedical Science (Biochemistry), and Australian Research Council Special Research Centre for the Molecular Genetics of Development, The University of Adelaide, Adelaide, South Australia, Australia; National Cancer Center, Japan

## Abstract

Neuronal Per-Arnt-Sim homology (PAS) Factor 4 (NPAS4) is a neuronal activity-dependent transcription factor which heterodimerises with ARNT2 to regulate genes involved in inhibitory synapse formation. NPAS4 functions to maintain excitatory/inhibitory balance in neurons, while mouse models have shown it to play roles in memory formation, social interaction and neurodegeneration. NPAS4 has therefore been implicated in a number of neuropsychiatric or neurodegenerative diseases which are underpinned by defects in excitatory/inhibitory balance. Here we have explored a broad set of non-synonymous human variants in NPAS4 and ARNT2 for disruption of NPAS4 function. We found two variants in NPAS4 (F147S and E257K) and two variants in ARNT2 (R46W and R107H) which significantly reduced transcriptional activity of the heterodimer on a luciferase reporter gene. Furthermore, we found that NPAS4.F147S was unable to activate expression of the NPAS4 target gene *BDNF* due to reduced dimerisation with ARNT2. Homology modelling predicts F147 in NPAS4 to lie at the dimer interface, where it appears to directly contribute to protein/protein interaction. We also found that reduced transcriptional activation by ARNT2 R46W was due to disruption of nuclear localisation. These results provide insight into the mechanisms of NPAS4/ARNT dimerisation and transcriptional activation and have potential implications for cognitive phenotypic variation and diseases such as autism, schizophrenia and dementia.

## Introduction

Basic Helix-Loop-Helix Per-Arnt-Sim homology (bHLH-PAS) proteins are signal regulated and/or tissue specific dimeric transcription factors involved in a diverse array of physiological and pathological functions [1]–[3]. They mediate processes such as the cellular response to hypoxia (Hypoxia Inducible Factors (HIF1α/HIF2α)) [2], the maintenance of circadian rhythms (Circadian Locomotor Output Cycles Kaput (CLOCK)) [4], the clearance of environmental pollutants (Aryl hydrocarbon Receptor (AhR)/Dioxin Receptor (DR)) [1], and appetite control (Single minded 1 (Sim1)) [5], [6]. The above bHLH-PAS transcription factors must heterodimerise with an obligate nuclear partner protein, Aryl hydrocarbon Receptor Nuclear Translator (ARNT/ARNT2) or Brain and Muscle ARNT-Like (BMAL1/BMAL2), to activate or repress gene expression[1]. Dimerisation is predominantly mediated through the conserved N-terminal bHLH and PAS repeat domains (PASA and PASB) to allow binding to asymmetric E-BOX-like elements in regulatory regions of target genes [7]–[9].

Neuronal PAS factor 4 (NPAS4) is a bHLH-PAS transcription factor whose expression and activity is tightly coupled with neuronal activity [10], [11]. Ischemia, seizure, neuronal depolarisation, and models of learning all rapidly and transiently increase expression of NPAS4 [10], [12], [13]. In response, NPAS4 activates a battery of genes to increase the number of inhibitory synapses, maintaining homeostasis of neuron activity [10], [14]. Not surprisingly, NPAS4 null mice are hyperactive, prone to seizures, and display several defects in social anxiety and cognitive impairments similar to those observed in autism and schizophrenia [10], [15]. NPAS4 null mice also have a much reduced life span due to extensive neurodegeneration, thought to be caused by glutamate neurotoxicity [16]. More recently, conditional deletion of NPAS4 in the CA3 region of the hippocampus in adult mice has shown it is required for contextual memory formation [11].

Neuropsychiatric disorders encompass a diverse array of phenotypes and while a strong genetic component has been suggested, the precise genes involved have been difficult to identify [15], [17], [18]. In addition, modifier or susceptibility genes are hypothesised to be required in concert with other mutations to drive aberrant neurological phenotypes [19], [20]. Recent reports from large scale whole genome and exome sequencing projects have highlighted the contribution that rare, *de novo* variants make to disease [19], [21]–[23]. Indeed, the large spectrum of phenotypes that underlie neuropsychiatric disease may reflect a multivaried nature of deleterious, rare genetic variants associated with disease. Many neuropsychiatric diseases, including schizophrenia and autism, are thought to have related defects which disrupt the balance between neuronal excitation and inhibition [24]–[27]. There is strong evidence from loss of function mutations associated with these diseases that synapse dysfunction may play a key role in disrupting this homeostatic balance [17], [28], [29]. In addition, glutamergic hyperexcitation, which may also arise from disruption of the excitatory/inhibitory balance or other neuronal stressors, has been linked to the progression of neurodegenerative disorders [30], [31].

Given NPAS4 is activated following neuronal activity to control the balance of excitatory and inhibitory synapses, and that defects in memory, social anxiety, age related neurodegeneration, hyperactivity and seizures are observed in NPAS4 null mice, NPAS4 has been implicated in a number of neuropsychiatric and neurodegenerative diseases [15], [16], [29], [32]. Here we sought to test whether human variants in NPAS4 or ARNT2 might compromise transcriptional function. We screened several variants listed in public databases and found examples where single amino acid changes in NPAS4 or ARNT2 resulted in mild or dramatic loss of function. We elucidated mechanisms of disrupted heterodimerisation or impaired nuclear localisation to attenuate activity of the NPAS4/ARNT2 complex. This study outlines a strategy to highlight and prioritise non-synonymous variants in human transcription factors which might then be screened in patient cohorts to investigate their possible contribution to disease.

## Materials and Methods

### Cloning and Cell Culture

pCMV-hNPAS4-mycFlag was purchased from Origene (Rockville,USA). NPAS4-mycFlag was subsequently subcloned into pENTR1a (Invitrogen) using SmaI/EcoRV. pEF-IRESpuro-hSIM1-2Myc and pEF-IRESneo-hARNT1, pEF-IRESneo-hARNT2, pEF-IRESpuro-hARNT1-3xFlag, pEF-IRESneo-hARNT2-3xFlag have been described previously [33]. pEFBOS-Gtwy and pcDNA-FRT/TO-Gtwy were created by inserting KpnI/EcoRV GtwyA from pLV410 [34] into either pEF-BOS or pcDNA-FRT/TO. NPAS4, ARNT2 and SIM1 variants were cloned using overlap extension PCR or Gibson Isothermal assembly essentially as described [35], [36] using Phusion proof reading polymerase (New England Biolabs). Primers were designed to incorporate the indicated human variants (Table S1) and the mutations were confirmed by sequencing. NPAS4 variants were either subcloned into pEFBOS-Gtwy or pcDNA5-FRT/TO-Gtwy plasmids using LR recombination (Invitrogen). pcDNA3.2-d2nucEGFP was created by first subcloning d2eGFP from pd2eGFP-T4 N1 (Clonetech) into pENTR1a using EcoRI/NotI, then D2nucEGFP was digested from pNSEN [37] with AgeI/HindIII and inserted into pENTR1a-d2eGFP. pcDNA3.2-DEST (Invitrogen) was then recombined with pENTR1a-d2nucEGFP by LR recombination.

Human Embryonic Kidney HEK293T (ATCC, CCL-3216), HEK293-TREX (Invitrogen) and Neuro2A (ATCC, CCL-131) cells were maintained in DMEM medium supplemented with 10% FCS, 1 mM L-glutamine, 100 units/ml penicillin, and 100 µg/ml streptomycin. HEK293-TREX stable cell lines were generated according to manufacturer instructions (Invitrogen). Briefly 6×10^5^ HEK293TREX cells were transfected with 2 µg of pOG44 and 200 ng of pcDNA5-FRT/TO-hNPAS4-mycFlag plasmid using Fugene6 (Roche). 48 hrs after transfection the cells were expanded and selected in 200 µg/ml Hygromycin B (Invitrogen) to generate stable cell lines. At least two independently derived WT NPAS4 cell lines and one cell line for each variant were used for BDNF exon I expression analysis. NPAS4 expression was induced with 1 µg/ml doxycyline for 24 hrs.

### Transient transfections and luciferase assays

HEK293T cells in 24 well plates were transiently transfected with a DNA cocktail containing 200 ng firefly luciferase reporter plasmid pML-6xCME-Luc or empty pML-Luc control [38], 50 ng each of ARNT1 or ARNT2 expression plasmid, pEF-IRESpuro-hSIM1-2myc, pEF-IRESpuro-hSIM2-2myc [39], [40] or pEFBOS-hNPAS4-mycFlag expression vectors and 200 pg of phRL-CMV Renilla luciferase reporter plasmid (Promega) using Fugene6 (Roche) according to the manufacturers' instructions. Plasmid concentrations were normalised using empty expression plasmids. After 24 or 48 hrs, relative luciferase activities for each bHLH/PAS variant were assayed using a DLR kit (Promega) and normalized to relative WT activity. For immunoprecipitations, 1 µg of pCI-eGFP, 2 µg of ARNT2 and 2 µg of NPAS4 expression plasmids were cotransfected into 5×10^5^ 293T cells using Fugene6 (Roche).

### RNA extraction, cDNA synthesis and quantitative real time PCR

HEK293-TREX cells were lysed in 500 µl trizol (Invitrogen) and RNA isolated. 2 µg of RNA was used in each reverse transcription reaction (Superscript III, Invitrogen) and cDNA was diluted 10 fold in 1X Tris-EDTA (TE) pH 8.0.for real time PCR. Real time PCR was performed in triplicate using Fast SYBR Green Master Mix (Applied Biosystems) on a StepOne Plus Real-time PCR system (Applied Biosystems) using primers specific to human BDNF exon I [41], NPAS4 [10] and RNA polymerase 2A [42] and spanning an intron where possible. BDNF gene induction by each NPAS4 variant was normalised to RNA polymerase 2A. Melt curves of PCR products were analysed to confirm a single amplicon and real time PCR results were analysed and ‘QGene’ analysis software [43].

### Immunoblotting and Immunoprecipitations

For immunoprecipitations, cells were washed twice in PBS and lysed in 20 mM HEPES, pH 8.0, 420 mM NaCl, 0.5% Igepal, 25% glycerol, 0.2 mM EDTA, 1.5 mM MgCl2, 1 mM DTT and protease inhibitors (Sigma). 100 µg of protein whole cell extract was diluted to 1 mg/ml in immunoprecipitation (IP) buffer (20 mM HEPES, pH 8.0, 150 mM NaCl, 150 mM KCl, 0.1% glycerol, 1 mM EDTA, 1 mM DTT and protease inhibitors) and incubated with 50 µL of BSA blocked Flag M2 resin (Sigma). The resin was washed twice with 1 mL of IP wash buffer (250 mM NaCl, 20 mM HEPES pH 8.0, 0.1% Igepal, and 1 mM EDTA) and the bound material boiled in 20 µl of SDS sample buffer. Input sample (10%) and immunoprecipitations were then run on 7.5%SDS-PAGE gels and transferred to nitrocellulose. Lysates from reporter gene assays were separated on 7.5% SDS-PAGE gel and transferred to nitrocellulose. Proteins were detected using the anti-ARNT2 (Santa Cruz), anti-FLAG (Sigma), anti-Myc (4A6, Upstate) and anti-α-Tubulin antibodies (MCA78G, Serotec). Primary antibodies were detected using horseradish peroxidise-conjugated secondary antibodies and visualised using chemiluminescence. Quantification of ARNT2 co-immunoprecipition band intensity was estimated using ImageLab software (BioRad).

### Immunofluorescence

pEF-IRESpuro-hARNT2-3xFlag or pEF-IRESpuro-hARNT2.R46W-3xFlag (50 ng) were cotransfected with 200 ng of pcDNA3.2-d2nucEGFP into HEK293T cells plated on Poly-D-Lysine (70–150 KDa; Sigma) coated coverslips using Fugene6 (Roche). After 24 hrs, Cells were fixed 4% Paraformaldehyde (PFA) for 20 mins, washed in PBS and permeablised using 2% Triton-X-100/PBS for 10 mins. The fixed cells were then blocked using horse serum, and ARNT2 detected using anti-Flag antibody (1∶1000; Sigma) and visualised using a TxRed-conjugated secondary antibody. Coverslips were mounted onto slides using Prolong Gold mounting medium containing DAPI (Invitrogen) and images taken using a Ziess deconvolution microscope. Images were then false coloured and overlayed using ImageJ imaging software [44].

### Homology modeling

Homology models were created in the ICM-Pro program suite [45] using the homology add-on [46], [47]. Individual subunits of the heterodimer were first created separately using the sequence for human NPAS4 (Uniprot number Q8IUM7) and for human Arnt2 (Uniprot number Q9HBZ2). A search for homologous structures in the Protein Data Bank was performed and the Clock-BMAL structure was the structure with the highest homology (PDB∶4F3L) [48]. The NPAS4 homology model was created using the CLOCK protein as a three-dimensional template and the ARNT2 homology model was created using BMAL as the three-dimensional molecular template. After creation of individual subunit models, they were both subjected to regularization and model refinement within ICM-Pro (energy minimization, optimization of geometry, and easing of clashing side-chains). Both subunits were then docked together using the CLOCK-BMAL structure to guide the docking. Further model refinement and regularization was then performed to ensure the integrity of the dimer interface. Finally, many loops were identified and subjected to loop modeling to improve clashing at the dimer interface using the ICM-Pro loop modeling utility [49]. Figures were created using PyMOL (The PyMOL Molecular Graphics System, Version 1.2r3pre, Schrödinger, LLC.).

### Statistical Analysis

Statistical significance was performed using GraphPad Prism 5 program for Windows (GraphPad Software Inc.) and evaluated by the Student's t test or ANOVA with the level of significance set at p<0.05. ANOVA statistical analysis was performed on log transformed data with Tukey's post hoc analysis. Data are expressed as mean ±SEM unless otherwise indicated.

## Results

**Figure 1:**
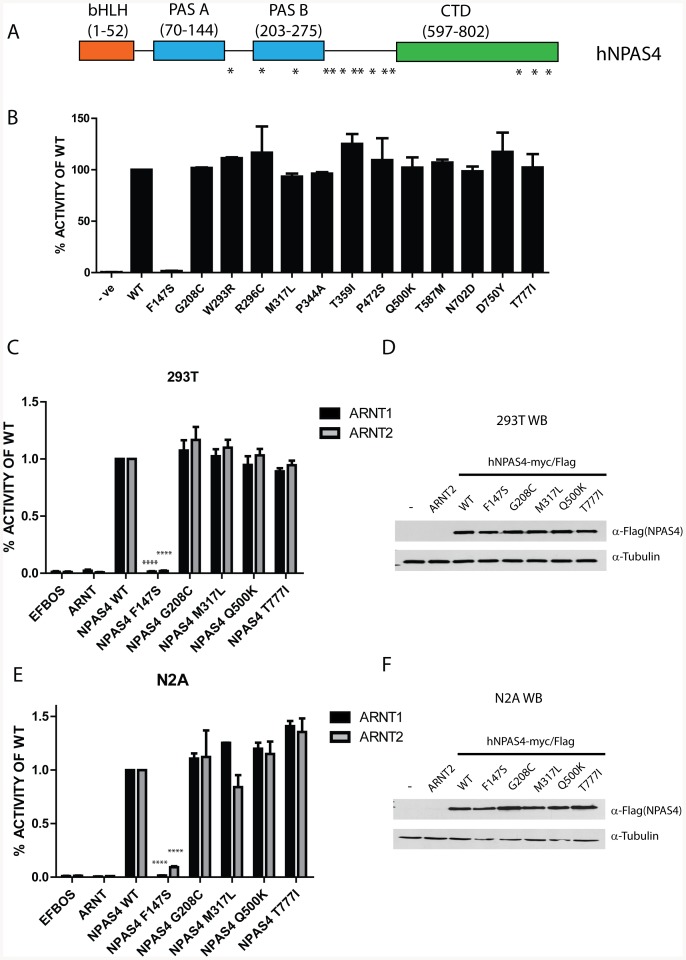
Functional Analysis of human NPAS4 Variants. A, Schematic of domains within NPAS4 showing positions of analysed non-synonymous variants. B, A screen of NPAS4-mycFlag variants using NPAS4/ARNT2 activation of a reporter gene (6xCME-Luc) in HEK293T cells transfected with NPAS4-MycFlag and ARNT2 expression vectors. Values represent average percentage activity of WT NPAS4-mycFlag ±SD of two independent experiments. C and E, NPAS4-MycFlag variant activation of the 6xCME-Luc reporter gene upon co-expression of ARNT1 or ARNT2 as the dimerisation partner in HEK293T cells (C) or Neuro2A cells (E). Data are mean percentage activity of WT NPAS4-mycFlag ±SEM of at least 3 independent experiments. Statistical significance was calculated using an ANOVA compared to WT NPAS4-mycFlag. Cell lysates from reporter assays in C and E were separated by SDS-PAGE and NPAS4-mycFlag protein detected by immunoblotting using α-Flag antibodies, with α-tubulin was used as a loading control. Representative western blots of NPAS4-mycFlag variants are shown for HEK293T cells (D) and Neuro2A cells (F). ****p<0.0001.

To investigate whether variants in NPAS4 would disrupt function we examined protein coding variants from the 1000 genomes project, the NHLBI exome sequencing project and the NCBI SNP database [50]–[52]. Initially we examined 13 non-synonymous NPAS4 variants, which represented all reported mismatch variants at the time we initiated this study, for their ability to activate a bHLH-PAS responsive luciferase reporter gene containing six repeats of a bHLH-PAS core binding element (pML-6xCME-Luc, [33]). The single nucleotide variants were spread throughout the protein sequence and spanned the C-terminal and PAS regions (Fig. 1A). Coexpression of wild type (WT) NPAS4 and ARNT2, but not ARNT2 alone, strongly activated the reporter gene (Fig. 1B). The overwhelming majority of variants were able to activate the reporter to a similar extent as WT NPAS4, however, variant F147S completely ablated the ability of NPAS4 to activate the reporter (Fig. 1B).

Although NPAS4 expression most strongly overlaps with ARNT2 within the brain, in biochemical experiments NPAS4 appears to show little bias between binding and activating transcription with ARNT1 or ARNT2 [53], [54]. To explore this further we then tested the ability of a subset of NPAS4 variants, including F147S, to activate the reporter gene in HEK293T cells in combination with either ARNT1 or ARNT2 as the dimerisation partner (Fig. 1C). NPAS4 was able to strongly activate the reporter when dimerising with either ARNT1 or ARNT2, although the F147S variant remained inactive irrespective of coexpression with ARNT1 or ARNT2 (P<0.0001, Fig. 1C). The loss of function NPAS4.F147S protein was expressed at similar levels and size (∼100 KDa) to that of WT NPAS4 or other variants by western blot analysis (Fig. 1D). Given that NPAS4 is a neuronal transcription factor, we were interested in establishing whether there were neuron specific effects not observed in HEK293T cells. Using Neuro2A (N2A) cells we found that WT NPAS4 was active with either ARNT1 or ARNT2, but the F147S variant again failed to activate the reporter gene (P<0.0001, Fig. 1E). We did not observe any consistent changes in any of the other variants or in the overall protein expression of these variants in N2A cells (Fig. E and F).

**Figure 2:**
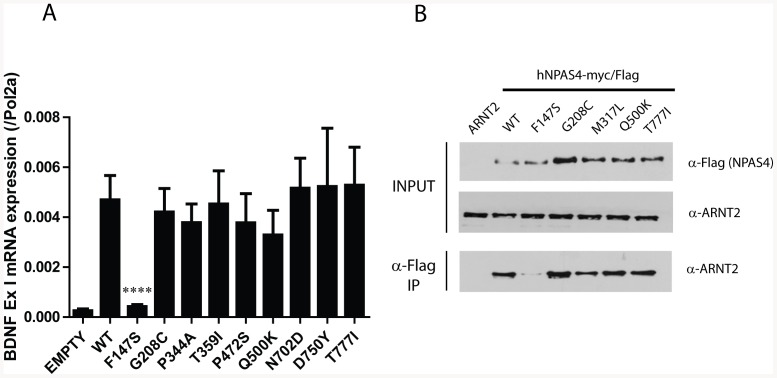
NPAS4 Variant F147S reduces dimerisation with ARNT2 and fails to activate *BDNF* expression. A, 293TREX cells containing site specific, stable integration of WT NPAS4-mycFlag, NPAS4-mycFlag variants or an empty vector were induced with 1 µg/ml doxycycline for 24 hrs and *Brain Derived Neurotrophic Factor* (*BDNF*) Exon I mRNA expression measured by quantitative real-time PCR and normalised to RNA Polymerase 2A. Data are mean ±SEM of at least 4 independent experiments; WT is an average of two independently derived cell lines and least 4 independent experiments. Statistical significance is calculated using an ANOVA compared to WT NPAS4-mycFlag, ****p<0.0001. B, Immunoblotting of whole cell extracts and α-Flag coimmunoprecipitates from HEK293T cells transiently transfected with NPAS4-MycFlag and ARNT2 expression constructs. α-Flag Abs used to detect NPAS4-MycFlag and α-ARNT2 Abs used to detect overexpressed ARNT2. Data are representative of three independent experiments.

To further characterise the mechanism of the loss of function NPAS4.F147S variant we generated HEK293-TREX inducible cell lines where NPAS4 or NPAS4 variant expression can be induced from a defined locus upon treatment with doxycycline. This allows for equivalent expression of NPAS4 variants, facilitating direct comparison of activities. NPAS4 has been previously shown to be a critical activator of *Brain Derived Neurotrophic Factor* (*BDNF*) following neuronal depolarisation [10], [41]. Furthermore, defective *BDNF* expression and function is also an important contributor to neurological disease [55], [56]. We therefore next investigated whether the NPAS4.F147S and a subset of variants spanning the C-terminus could induce *BDNF* exon I expression using 293TREX cells. Overexpression of WT NPAS4 and the C-terminal variants were able to strongly induce the expression of *BDNF* exon I, however, there was a ∼90% reduction in the ability of F147S to induce *BDNF* exon I expression (Fig. 2A). Similar reduction in the ability of NPAS4.F147S to activate *BDNF* exon I expression was found in transient transfection experiments where ARNT2 was coexpressed (Fig. S1). We did not observe any significant differences in the ability of any other NPAS4 variants to activate *BDNF I* expression (Fig. 2A).

**Figure 3:**
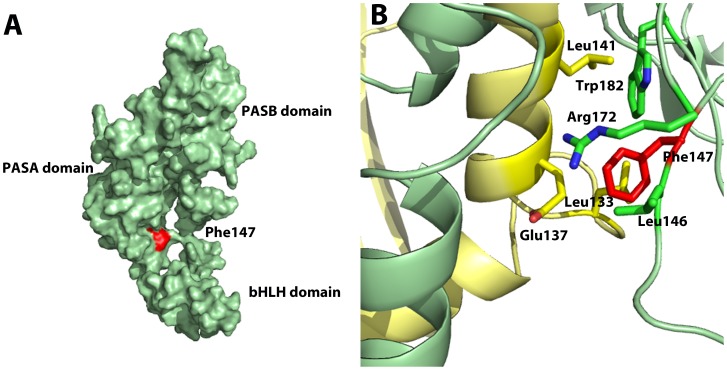
Homology model highlighting residue Phe147 of NPAS4. NPAS4 is colored green and ARNT2 is colored yellow. A) The NPAS4 subunit is depicted as a surface representation. The location of Phe147 is shown by red color. B) The NPAS4-ARNT2 heterodimer is depicted as a ribbons diagram. Phe147 is shown at the interface with side chain shown as sticks colored red, together with amino acids within 3.5 angstroms. Phe 147 is part of a hydrophobic pocket formed with ARNT2 residues (Leu 133, Leu 141) and NPAS4 residues (Leu 146, Trp 182).

**Figure 4:**
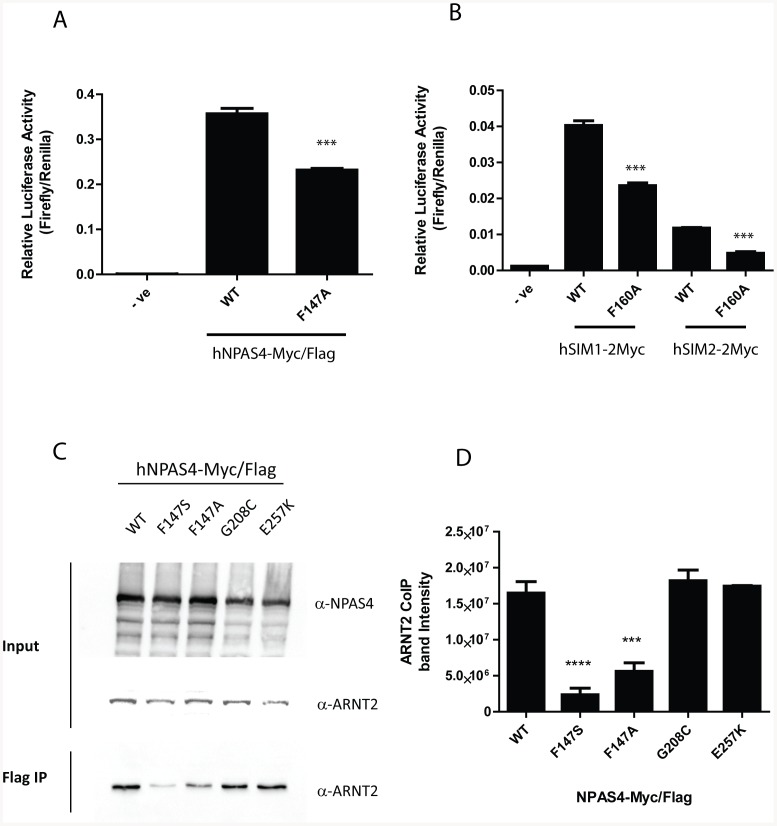
NPAS4.F147A, SIM1.F160A, and SIM2.F160A show reduced activities on a reporter gene. A, Comparison of WT NPAS4-mycFlag and NPAS4.F147A-mycFlag activities on reporter gene 6xCME-Luc in HEK293T cells transfected with NPAS4-MycFlag and ARNT2 expression vectors. B, Comparison of WT SIM-2myc proteins with SIM1.F160A-2myc and SIM2.F160A-2myc variants on reporter gene 6xCME-Luc in HEK 293T cells cells transfected with SIM1-2Myc, SIM2-2Myc and ARNT2 expression vectors. C. Immunoblotting of whole cell extracts and α-Flag coimmunoprecipitates from HEK293T cells transiently transfected with NPAS4-MycFlag. α-Flag Abs used to detect NPAS4-MycFlag and α-ARNT2 Abs used to detect endogenous ARNT2. D. ARNT2 coimmunopreciption band intensity quantitation of 3 independent α-Flag coimmunoprecipitation experiments. Data are mean relative luciferase activities ±SEM of 3 experiments. Statistical significance is calculated using an unpaired two tailed students t-test (A and B) or ANOVA (D) compared to WT.***p<0.001, ****p<0.0001.

The NPAS4.F147S variation was located towards the end of the PASA domain in a region where amino acids important for dimerisation have been previously identified (Fig.S2A and Fig. 1A) [33], [57]. We were therefore interested to test whether the F to S conversion could disrupt dimerisation between NPAS4 and ARNT2. We used co-immunoprecipitation to show that NPAS4.F147S failed to form a heterodimer with ARNT2 (Fig. 2B). Other NPAS4 variants, which showed near wild type activities on the reporter gene (Fig 1C), showed similar ARNT2 co-immunoprecipitation to wild type NPAS4. Homology modelling of NPAS4 and ARNT2 using the crystal structure for CLOCK and BMAL as a template revealed F147 to lie on the surface (Fig. 3A) and position at the proposed dimerisation interface between NPAS4 and ARNT2 (Fig. 3B) [48]. The phenylalanine residue appears to be in close proximity to several hydrophobic residues in ARNT2 and may make contacts within this interface to promote dimerisation (Fig. 3A). Substitution of the large, hydrophobic phenylalanine with a small, polar serine may therefore explain the loss in dimerisation. We hypothesised that substitution of F147 with an alanine may partially disrupt function by removing the larger phenylalanine residue, but maintaining hydrophobicity. We therefore tested this mutant and found NPAS4.F147A significantly (P<0.001) decreased reporter gene activity to approximately 65% of wt, supporting our hypothesis (Fig. 4A). Furthermore, mutation of the corresponding phenylalanine residue to alanine in related bHLH/PAS proteins SIM1 (SIM1.F160A) and SIM2 (SIM2.F160A) also significantly (P<0.001) reduced reporter gene activities to approximately 60% and 40%, respectively, suggesting this mechanism of dimerisation may be shared among bHLH-PAS transcription factors (Fig. 4B). NPAS4.F147S and NPAS4.F147A also showed significantly (P<0.0001 and P<0.001, respectively) reduced dimerization with endogenous ARNT2 in co-immunoprecipition experiments compared to WT NPAS4 (Fig. 4C and 4D). Dimerisation with ARNT2 appeared to be greater with NPAS4.F147A than NPAS4.F147S, consistent with reporter gene data. Alignment of the human bHLH-PAS transcription factors revealed that the phenylalanine at this position was conserved among all the bHLH-PAS transcription factors, suggesting that this may represent an important amino acid for a conserved mode of dimerisation (Figure S2A).

**Figure 5:**
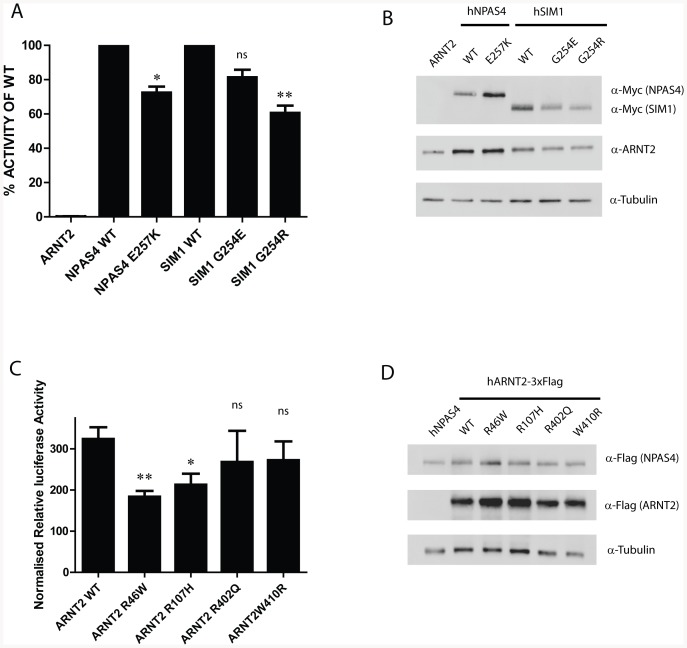
Partial loss of activity variants found in NPAS4, SIM1 and ARNT2. A, Variants of NPAS4-mycFlag, and SIM1-2myc were coexpressed with ARNT2 and assessed for activation of reporter gene 6xCME-Luc in HEK293T cells. Data are presented as average percentage activities (±SEM of 3 independent experiments) of the relevant WT heterodimers which have been normalised 100%. B, Western blot of reporter assay lysates from A to verify expression of hSIM1-2myc and hNPAS4-mycFlag (using α-Myc antibodies), hARNT2 (α-ARNT2 antibodies) and tubulin (α-tubulin antibodies). C, NPAS4-mycFlag activation of reporter gene 6xCME-Luc in combination with the indicated ARNT2-3xFlag variants in HEK293T cells. Data are presented as average percentage activities (±SEM of 3 independent experiments) of WT NPAS4-mycFlag/ARNT2-3xFlag heterodimer which has been normalised to 100%. D, Western blot of reporter assay lysates from C to detect expressed hNPAS4-MycFlag and hARNT2-3xFlag with α-flag antibodies, or tubulin using α-tubulin antibodies. Statistical significance was calculated using an ANOVA comparing relative luciferase activities to WT, * p<0.05, **p<0.01.

Recently we have found a number of Loss of Function (LoF), non-synonymous variants of SIM1 in a cohort of children displaying early onset, morbid obesity[58], [59]. A number of these LoF SIM1 variants were clustered around PASA and PASB domains. In addition, a number of residues within a homologous region of PASB have been shown to be important for dimerisation of CLOCK and BMAL [48]. New variants from recent sequencing projects became available during this study and we identified one additional variant in NPAS4 (E257K) and two variants in SIM1 (G254E and G254R) which lay within this region of clustered LoF mutations (Figure S2B). As these variants replaced well conserved residues and had drastically altered side chain chemistry, we reasoned they might alter protein activities. Using luciferase reporter gene assays we found that the NPAS4 E257K variant significantly (p<0.05) reduced activity to ∼70% when compared to WT (Fig. 5A). The reduced reporter activity observed with the NPAS4.E257K mutation did not appear to be due a reduction in dimerisation with ARNT2 (Fig. 4C and 4D) or protein expression (Fig. 5B). SIM1 G254E and G254R both reduced luciferase reporter activity, however only SIM1.G254R reached statistical significance (G245E, p>0.05; G254R, p<0.01). The SIM1.G254R variant reduced reporter activity to ∼60% of WT, which may in part be due to a reduction in SIM1 protein expression (Fig 5B).

**Figure 6:**
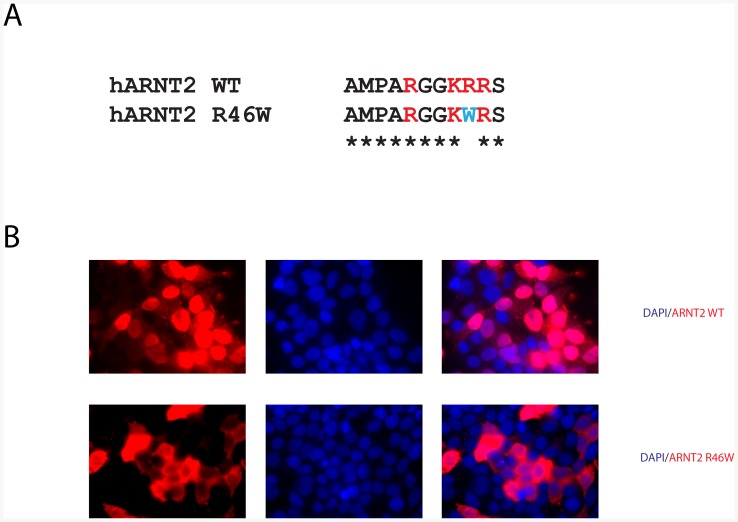
Variant R46W in ARNT2 disrupts nuclear localisation. A, Alignment of the N-terminal basic residues of ARNT1 nuclear localisation sequence with WT ARNT2 and ARNT2.R46W. B, Immunofluorescence of HEK293T cells transfected with either WT ARNT2-3xFlag or ARNT2.R46W-3xFlag expression vectors using α-Flag antibodies (Red) and nuclei stained with DAPI (Blue).

Since our data revealed certain variants in NPAS4 to disrupt the transcriptional output from NPAS4, we then predicted that variants in ARNT2 might indirectly disrupt NPAS4 dependent transcriptional output. We therefore examined single nucleotide variants in ARNT2, concentrating on those that invoked dramatic changes in side chain chemistry within functional N-terminal domains where LoF variants were likely to occur. We selected and tested four variants in ARNT2, R46W, R107H, R402Q and W410R, for their ability to activate the 6xCME-Luciferase reporter gene in combination with NPAS4. Expression of NPAS4 alone was unable to activate the reporter, however when ARNT2 was coexpressed we observed strong activation (Fig. 5C). Both ARNT2.R46W and ARNT2.R107H significantly (P<0.01 and P<0.05, respectively) reduced activation of the reporter to 55% and 65% compared to WT ARNT2, respectively. No significant differences were observed with ARNT2.R402Q or ARNT2.W410R (Fig. 5C). Expression of these ARNT2 variants was comparable to WT ARNT2 (Fig. 5D). Previously, it has been shown that constitutively nuclear localisation of ARNT1 is controlled by a small N-terminal bi-partite nuclear localisation sequence (NLS) [60]. Alignment of ARNT1 and ARNT2 revealed that ARNT2 also shared this NLS and that R46W is within a set of basic residues that comprise the NLS (Fig. 6A). We hypothesised that the LoF exhibited by the ARNT2.R46W variant may be due to loss of nuclear localisation and therefore performed immunocytochemistry on Flag tagged WT or ARNT2.R46W expressed in HEK293T cells (Fig. 6B). As expected, WT ARNT2 was constitutively nuclear in almost all transfected cells, whereas ARNT2.R46W was cytoplasmic in the majority of transfected cells (Fig. 6B). We conclude that weak reporter gene activity of variant R46W is therefore due to deficient nuclear import. The ARNT2.R107H variant mildly impaired activity. R107 lies within a proposed helix of the bHLH domain of ARNT2 and mapping this amino acid on the homology model of ARNT2 predicts that R107 is also surface exposed, positioned at the interface between NPAS4 and ARNT2. The R to H transition may slightly weaken dimerisation between NPAS4 and ARNT2 although this was not evident from co-immunoprecipition experiments with NPAS4 (data not shown).

In summary, we have analysed a series of natural human variants in NPAS4 and ARNT2 and discovered a subset which compromise transcriptional activation. LoF variants are clustered within domains with known roles in dimerisation and nuclear localisation. Taken together, this data suggests that while many single amino acid variants in the NPAS4 and ARNT2 transcriptional complex may not compromise activity, distinct sites in the N-terminal functional domains are sensitive to alteration and warrant further investigation into possible links with neurological disorders.

## Discussion

Protein coding variants are estimated to be abundantly generated in healthy individuals, but can also be important contributors to disease [51], [61]. The notion of *de novo* or rare variant generation as drivers of complex diseases such as autism is being increasingly appreciated [21], [23]. Although large scale sequencing projects have produced bioinformatic estimations for the contribution of natural protein coding variation to protein dysfunction, this has not been addressed experimentally [61]. Intellectual disability, autism and schizophrenia have been linked to mutations in synaptic proteins, some of which have been shown to underpin defects in synapse structure, function or homeostatic balance in animal models [28], [62]–[64]. It has therefore been hypothesised that alterations to activity-dependent neuronal signalling may disrupt neuronal homeostasis as a common mechanism in neuropsychiatric disease [17], [29].

NPAS4 is a transcription factor critical for maintaining homeostatic balance of excitation/inhibition within neurons and has been implicated in several neurological diseases [11], [15], [29], [32]. Mice lacking NPAS4 exhibit many hallmarks of both neuropsychiatric and neurodegenerative diseases, but as yet this transcription factor has not been linked to any human disorder. While this may seem surprising, many of these diseases are multifaceted, do not manifest until later in life and may be dependent upon additional environmental or genetic susceptibility factors.

The rapid expansion in genome sequencing data has recently provided a wealth of human protein coding variants with little or no associated phenotypic information. Here we tested the effects of several human protein coding variants in NPAS4 and ARNT2 on activity of the NPAS4/ARNT2 transcription factor complex. We initially screened all NPAS4 non-synonymous coding variants available in the dbSNP database at the outset of the study, in the context of an NPAS4/ARNT2 heterodimer. We found NPAS4.F147S to have a near complete loss of function on both a reporter gene and the endogenous *BDNF* target gene, which was a consequence of disrupted dimerisation with ARNT2. In contrast, the other twelve NPAS4 variants we screened failed to significantly reduce induction of the reporter gene and/or *BDNF*. We note that the majority of these variants (8/13) lie in a region spanning 300 amino acids following PASB (Fig 1A), which is predicted to be largely unstructured and seemingly tolerant for variation. As SNP databases expanded, we tested variants in NPAS4 and ARNT2 which we predicted might be prone to altered activity due to position in key domains and distinct change in amino acid side chain chemistry. This led to the discovery that variant E257K in NPAS4 had a mild attenuation of activity (∼70% of WT), an observation recapitulated with similarly located variants (G254E, G254R) within the related bHLH-PAS SIM1 transcription factor (Fig 5A). In addition, we successfully predicted and verified the R46W variant in ARNT2, which lies within a nuclear localisation sequence, to have significantly weaker activity than wild type due to attenuated nuclear uptake.

We recently found that rare variants within the bHLH-PAS transcription factor SIM1 were present in severely obese children or adults with hyperphagic Prada-Willi Like syndrome [58], [59]. While non-synonymous variants with partial loss of function were found throughout SIM1, those with the most severe effects on activity were clustered within N-terminal domains important for dimerisation and DNA binding. Furthermore, using a reverse bacterial two-hybrid system, we have previously defined several amino acids within the PASA domain of bHLH-PAS heterodimers that are important for dimerisation [33]. These tend to be clustered within a conserved β-sheet towards the end of PASA, where the NPAS4 variant F147 resides (Figure S2A). Using homology modelling of NPAS4 and ARNT2 based on the CLOCK/BMAL heterodimer crystal structure we confirmed that F147 likely lies at the NPAS4/ARNT2 PASA dimer interface. Furthermore, maintenance of hydrophobicity by substitution with alanine at this position only partially disrupted reporter activation and dimerisation (Fig. 4A, Fig. 4C, and Fig. 4D), suggesting that the presence of a large hydrophobic residue is required for optimal dimerisation. Sequence alignments of human bHLH-PAS transcription factors also revealed that the F147 residue is highly conserved at the corresponding position in other bHLH-PAS transcription factors (Figure S2A). Consistent with this notion, replacing this phenylalanine with alanine in SIM1 and SIM2 also led to partial loss of function of the SIM/ARNT2 heterodimers (Fig 4B). Within the CLOCK/BMAL structure the A'α helix of CLOCK PASA make strong interactions with the β-sheet faces of BMAL, and conversely the BMAL A'α helix makes contacts with the β-sheet face of CLOCK to mediate dimerisation [48]. This helps explain the lack of NPAS4/ARNT2 interaction for NPAS4.F147S, and also why a screen for PAS A mutations in ARNT which inhibit AhR/ARNT dimerisation recovered modifications within the surface exposed β-sheet face of ARNT [33]. Taken together this supports the notion of F147 being a key residue for dimerisation among bHLH-PAS transcription factors and outlines the importance of this region for intermolecular interaction and heterodimerisation between PASA domains [33], [48].

This study of human NPAS4 and ARNT2 non-synonymous variants is the first screen that we are aware of that assess the specific activities of natural transcription factor variants. While protein coding variants in NPAS4 have yet to be associated with a human disorder, the region surrounding NPAS4 has been found to be deleted in a boy with intellectual disability [65], and a region encompassing NPAS4 has been found to be associated with bipolar disorder [20]. Effects of the variants we tested includes near complete loss of function of the NPAS4/ARNT2 heterodimer (NPAS4.F147S) and partial loss of function (NPAS4.E257K, ARNT2.R46W and ARNT2.R107H). While we cannot assess the impact of these variants on phenotype, experiments using NPAS4 deficient mice suggest affected phenotypes may encompass low severity mild memory deficits and social interaction deficits, through to more severe conditions of epilepsy, schizophrenia or age related neurodegeneration [10], [11], [15], [16]. Our results now seem to warrant targeted sequencing of NPAS4 in cohorts of neuropsychiatric and/or dementia patients.

Finally, the number of new variants identified from ongoing sequencing projects has significantly expanded since the initiation of this study. The methods outlined here provide a rapid and efficient way of testing for loss of function or weak activity variations in bHLH-PAS transcription factors and can be used to examine other disease related bHLH-PAS transcription factors such as SIM1 or HIF1α, as additional variants from new sequencing projects becomes available.

## Supporting Information

Figure S1 **NPAS4.F147S has reduced ability to induce BDNF exon I mRNA expression in HEK293T cells.** HEK293T cells transiently transfected with NPAS4-MycFlag, ARNT2, or control expression vectors. Brain Derived Neurotrophic Factor (BDNF) exon I mRNA expression measured by quantitative real-time PCR and normalised to RNA Polymerase 2A. Data are mean ±SEM of 3 independent experiments. Statistical significance is calculated using an ANOVA compared to WT NPAS4-mycFlag. ** p<0.01, ***p<0.001.
(TIF)

Figure S2 **Conservation of the PAS domains of bHLH-PAS transcription factors using multiple sequence alignments.** Multiple sequence alignments of selected regions of A) basic helix loop helix (bHLH) and PASA regions or B) the PASB regions of human bHLH-PAS transcription factors. Conserved residues of selected variants (ARNT2 R107, NPAS4.F147, NPAS4.G208, NPAS4.E257 and SIM1 G254) in this study are highlighted in red, activity deficient variants from CLOCK/BMAL structure [1], ARNT/AhR [2], [3] or SIM1 [4], [5] are in magenta.
(DOCX)

Table S1 **Oligonucleotides used for cloning bHLH-PAS variants.**
(DOCX)
