# Supplementary figures and images for: Human Variants in the Neuronal Basic Helix-Loop-Helix/Per-Arnt-Sim (bHLH/PAS) Transcription Factor Complex NPAS4/ARNT2 Disrupt Function
Source: PLoS One. 2014 Jan 17;9(1):e85768. doi: 10.1371/journal.pone.0085768 (PMC3894988; doi:10.1371/journal.pone.0085768)

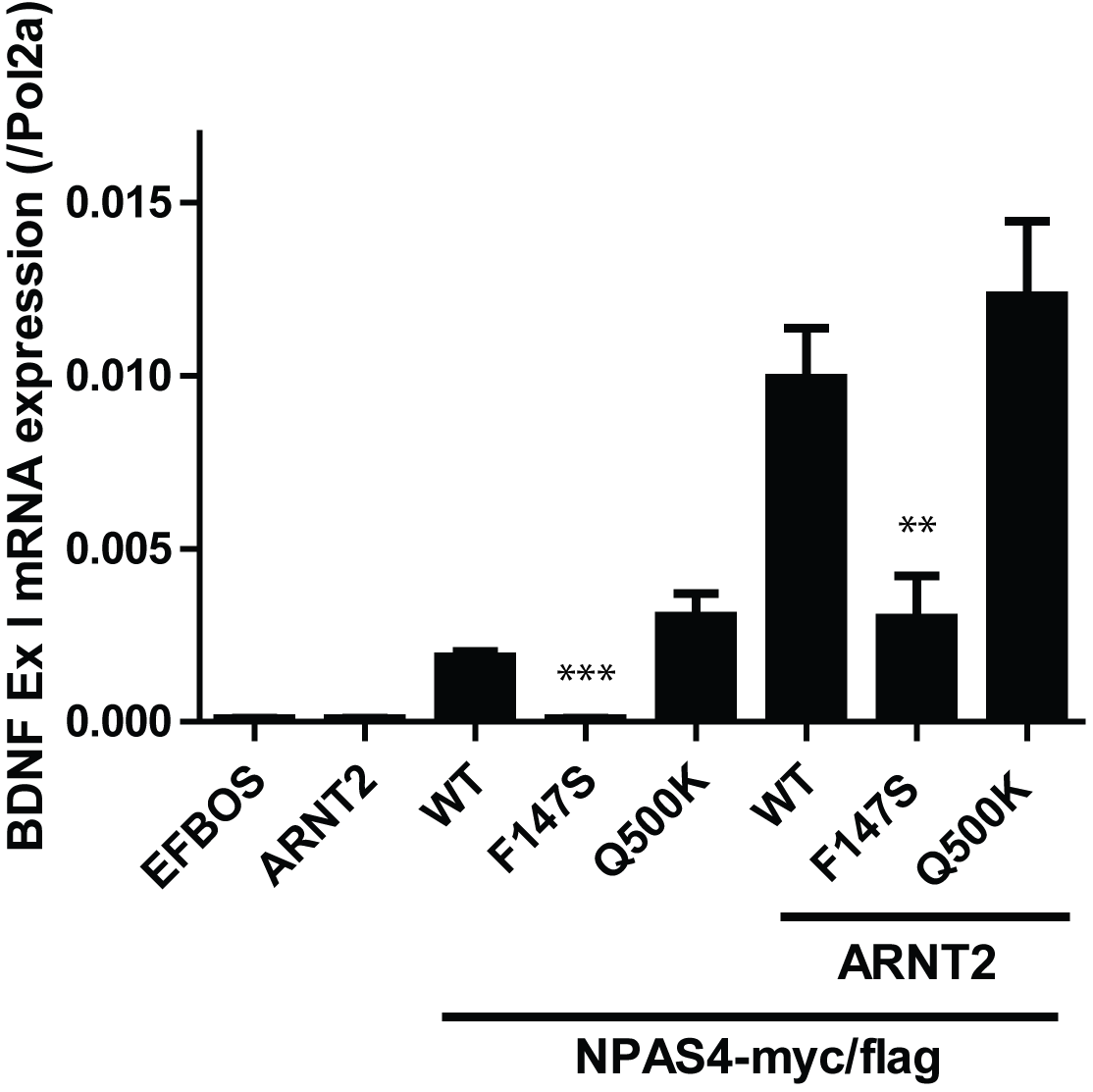

Supplement: Figure S1 — NPAS4.F147S has reduced ability to induce BDNF exon I mRNA expression in HEK293T cells. HEK293T cells transiently transfected with NPAS4-MycFlag, ARNT2, or control expression vectors. Brain Derived Neurotrophic Factor (BDNF) exon I mRNA expression measured by quantitative real-time PCR and normalised to RNA Polymerase 2A. Data are mean ±SEM of 3 independent experiments. Statistical significance is calculated using an ANOVA compared to WT NPAS4-mycFlag. ** p<0.01, ***p<0.001. (TIF) [file pone.0085768.s001.tif]
