# Supplementary material for: Human Variants in the Neuronal Basic Helix-Loop-Helix/Per-Arnt-Sim (bHLH/PAS) Transcription Factor Complex NPAS4/ARNT2 Disrupt Function
Source: PLoS One. 2014 Jan 17;9(1):e85768. doi: 10.1371/journal.pone.0085768 (PMC3894988; doi:10.1371/journal.pone.0085768)
Supplement: Figure S2 — Conservation of the PAS domains of bHLH-PAS transcription factors using multiple sequence alignments. Multiple sequence alignments of selected regions of A) basic helix loop helix (bHLH) and PASA regions or B) the PASB regions of human bHLH-PAS transcription factors. Conserved residues of selected variants (ARNT2 R107, NPAS4.F147, NPAS4.G208, NPAS4.E257 and SIM1 G254) in this study are highlighted in red, activity deficient variants from CLOCK/BMAL structure [1], ARNT/AhR [2], [3] or SIM1 [4], [5] are in magenta. (DOCX) [file pone.0085768.s002.docx]

A.

**bHLH**

**H**

**PAS A**

hNPAS1 PLPGAISSQLDKASIVRLSVTYLR 96 SLVQERSFFVRMKSTLTKRG 259 LGHTLPPAPLAELPLHGHMI 305

hNPAS3 PLPAAITSQLDKASIIRLTISYLK 102 DNTLERSFFIRMKSTLTKRG 275 VAHALPPPTINEVRIDCHMF 333

hSIM1 PLPSAITSQLDKASIIRLTTSYLK 51 EYEIERSFFLRMKCVLAKRN 172 VGHSLPPSAVTEIKLHSNMF 232

hSIM2 PLPSAITSQLDKASIIRLTTSYLK 51 EYEIERSFFLRMKCVLAKRN 172 VGQSLPPSAITEIKLYSNMF 232

hHIF1a PLPHNVSSHLDKASVMRLTISYLR 68 EQNTQRSFFLRMKCTLTSRG 179 ICEPIPHPSNIEIPLDSKTF 242

hHIF2a PLPHSVSSHLDKASIMRLAISFLR 65 DMSTERDFFMRMKCTVTNRG 178 MCEPIQHPSHMDIPLDSKTF 244

hARNT1 PTCSALARKPDKLTILRMAVSHMK 140 CMGSRRSFICRMRCGSSSVD 275 QVTSSPNCTDMSNVCQPTEF 363

hARNT2 PTCSALARKPDKLTILRMAVSHMK 114 CMGSRRSFICRMRCGNAPLD 249 QVTSSPVCMDMNGMSVPTEF 336

hBMAL1 PTCNAMSRKLDKLTVLRMAVQHMK 80 CSGARRSFFCRMKCNRPSVK 216 HSHVVPQPVNGEIRVKSMEY 287

hBMAL2 PQCNPMARKLDKLTVLRMAVQHLR 121 YSGSRRSFFCRIKSCKISVK 257 QPYIVPQ-NSGEINVKPTEF 334

hCLOCK PGN---ARKMDKSTVLQKSIDFLR 82 KSKNQLEFCCHMLRGTIDPK 190 ATPQFIKEMCTVEEPN-EEF 276

hNPAS2 PGN---TRKMDKTTVLEKVIGFLQ 55 KSDSDLEFYCHLLRGSLNPK 206 ATPQFLKEMCIVDEPL-EEF 251

hNPAS4 PLAEADKVRLSYLHIMSLACIYTR 51 ALDTDRLFRCRFNTSKSLRR 159 RPRPGPGPGPGPASLFLAMF 217

hAhR PFPQDVINKLDKLSVLRLSVSYLR 78 SPLMERCFICRLRCLLDNSS 232 IATPLQPPSILEIRTKNFIF 287

* : . :: . : * ::

**Loop Helix** **Gβ                                 Linker Aβ**

B.

**PAS B**

hNPAS1 GLTILACESRVSDHMDLGPSELVGR-SCYQFVHGQDATRIRQSHVDLLDKG--QVMTGYY 368

hNPAS3 DLNIIYCENRISDYMDLTPVDIVGK-RCYHFIHAEDVEGIRHSHLDLLNKG--QCVTKYY 396

hSIM1 DMKLIFLDSRVAELTGYEPQDLIEK-TLYHHVHGCDTFHLRCAHHLLLVKG--QVTTKYY 295

hSIM2 DLKLIFLDSRVTEVTGYEPQDLIEK-TLYHHVHGCDVFHLRYAHHLLLVKG--QVTTKYY 295

hHIF1a DMKFSYCDERITELMGYEPEELLGR-SIYEYYHALDSDHLTKTHHDMFTKG--QVTTGQY 305

hHIF2a DMKFTYCDDRITELIGYHPEELLGR-SAYEFYHALDSENMTKSHQNLCTKG-—QVVSGQY 307

hARNT1 EGIFTFVDHRCVATVGYQPQELLGK-NIVEFCHPEDQQLLRDSFQQVVKLKG-QVLSVMF 427

hARNT2 DGIITFVDPRCISVIGYQPQDLLGK-DILEFCHPEDQSHLRESFQQVVKLKG-QVLSVMY 401

hBMAL1 DGKFVFVDQRATAILAYLPQELLGT-SCYEYFHQDDIGHLAECHRQVLQTRE-KITTNCY 403

hBMAL2 NGKFVYVDQRATAILGYLPQELLGT-SCYEYFHQDDHNNLTDKHKAVLQSKE-KILTDSY 350

hCLOCK EWKFLFLDHRAPPIIGYLPFEVLGT-SGYDYYHVDDLENLAKCHEHLMQYG--KGKSCYY 339

hNPAS2 EWKFLFLDHRAPPIIGYLPFEVLGT-SGYDYYHIDDLELLARCHQHLMQFG--KGKSCCY 314

hNPAS4 DLALLDISESVLIYLGFERSELLCK-SWYGLLHPEDLAHASAQHYRLLAESGDIQAEMVV 282

hAhR DFTPIGCDAKGRIVLGYTEAELCTRGSGYQFIHAADMLYCAESHIRMIKTG--ESGMIVF 351

. :: * * . :.

**Bβ** **Cα** **Dα           ** **Eα             ** **Fα                 Gβ**

1. Huang N, Chelliah Y, Shan Y, Taylor CA, Yoo SH, et al. (2012) Crystal structure of the heterodimeric CLOCK:BMAL1 transcriptional activator complex. Science 337: 189-194.

2. Hao N, Whitelaw ML, Shearwin KE, Dodd IB, Chapman-Smith A (2011) Identification of residues in the N-terminal PAS domains important for dimerization of Arnt and AhR. Nucleic acids research 39: 3695-3709.

3. Sun W, Zhang J, Hankinson O (1997) A mutation in the aryl hydrocarbon receptor (AHR) in a cultured mammalian cell line identifies a novel region of AHR that affects DNA binding. The Journal of biological chemistry 272: 31845-31854.

4. Bonnefond A, Raimondo A, Stutzmann F, Ghoussaini M, Ramachandrappa S, et al. (2013) Loss-of-function mutations in SIM1 contribute to obesity and Prader-Willi-like features. The Journal of clinical investigation 123: 3037-3041.

5. Ramachandrappa S, Raimondo A, Cali AM, Keogh JM, Henning E, et al. (2013) Rare variants in single-minded 1 (SIM1) are associated with severe obesity. The Journal of clinical investigation 123: 3042-3050.
