# Supplementary material for: Human Variants in the Neuronal Basic Helix-Loop-Helix/Per-Arnt-Sim (bHLH/PAS) Transcription Factor Complex NPAS4/ARNT2 Disrupt Function
Source: PLoS One. 2014 Jan 17;9(1):e85768. doi: 10.1371/journal.pone.0085768 (PMC3894988; doi:10.1371/journal.pone.0085768)
Supplement: Table S1 — Oligonucleotides used for cloning bHLH-PAS variants. (DOCX) [file pone.0085768.s003.docx]

| Primer Name | Sequence |
| --- | --- |
| ARNT2 F | 5’ GGCGCAGACGGAACAAGATG 3’ |
| ARNT2 R | 5’ CCTTGGGAGTAGATCTGCTG 3’ |
| hARNT2 R46W F | 5' CCTGCCCGTGGAGGAAAGTGGAGATCTGGAATGGACTTCGATGATGAAGA 3' |
| hARNT2 R46W R | 5' TCTTCATCATCGAAGTCCATTCCAGATCTCCACTTTCCTCCACGGGCAGG 3' |
| hARNT2 R107H F | 5' CCAGACAAGCTCACCATCCTCCATATGGCCGTCTCGCACATGAAG 3' |
| hARNT2 R107H R | 5' CTTCATGTGCGAGACGGCCATATGGAGGATGGTGAGCTTGTCTGG 3' |
| hARNT2 R402Q F | 5' CCAAGTCCTGTCGGTCATGTATCAATTTCGAACCAAGAACCGGGAG 3' |
| hARNT2 R402Q R | 5' CTCCCGGTTCTTGGTTCGAAATTGATACATGACCGACAGGACTTGG 3' |
| hARNT2 W410R F | 5' CGCACCAAGAACCGGGAGCGCATGCTGATCCGCACCAGCAGCT 3' |
| hARNT2 W410R R | 5' AGCTGCTGGTGCGGATCAGCATGCGCTCCCGGTTCTTGGTGCG 3' |
| hNPAS4 F147A F | 5' TGCCCTGGACACTGATCGGCTAGCCCGCTGCCGCTTCAAC 3' |
| hNPAS4 F147A R | 5' GTTGAAGCGGCAGCGGGCTAGCCGATCAGTGTCCAGGGCA 3' |
| hNPAS4 F147S F | 5' CTGGACACTGATCGCCTCTCCCGCTGCAGATTCAACAC 3' |
| hNPAS4 F147S R | 5' GTGTTGAATCTGCAGCGGGAGAGGCGATCAGTGTCCAG 3' |
| hNPAS4 G208C F | 5' CCTGGCCCTGGCCCTTGCCCTGCTAGCCTCTTCC 3' |
| hNPAS4 G208C R | 5' GGAAGAGGCTAGCAGGGCAAGGGCCAGGGCCAGG 3' |
| hNPAS4 E257K F | 5' GGTATGGACTGCTGCACCCCAAAGATCTGGCCCACGCTTCTGC 3' |
| hNPAS4 E257K R | 5' GCAGAAGCGTGGGCCAGATCTTTGGGGTGCAGCAGTCCATACC 3' |
| hNPAS4 W293R F | 5' CAAGACTGGAGGCTGGGCGCGCATTTACTGCCTGTTATACTCAGAAGGTC 3' |
| hNPAS4 W293R R | 5' GACCTTCTGAGTATAACAGGCAGTAAATGCGCGCCCAGCCTCCAGTCTTG 3' |
| hNPAS4 C296R F | 5' AGGCTGGGCATGGATTTACGGTCTACTATACTCAGAAGGTCCAGAGGGA 3' |
| hNPAS4 C296R R | 5' TCCCTCTGGACCTTCTGAGTATAGTAGACCGTAAATCCATGCCCAGCCT 3' |
| hNPAS4 M317L F | 5' CCCAATCAGTGACCTCGAGGCCTGGAGCCTCCGC 3' |
| hNPAS4 M317L R | 5' GCGGAGGCTCCAGGCCTCGAGGTCACTGATTGGG 3' |
| hNPAS4 P344A F | 5’ ATGCTGGCAAGCTTCCCTGAAAACATTCTTTCCCAG 3’ |
| hNPAS4 P344A R | 5’ CAGGGAAGCTTGCCAGCATGGTCGGAGTGCC 3’ |
| hNPAS4 M359I F | 5’ AAGAGTGCTCGAGCATTAACCCACTCTTCACCGC 3’ |
| hNPAS4 M359I R | 5’ GGTTAATGCTCGAGCACTCTTCCTGGGAAAGAATGTTTTC 3’ |
| hNPAS4 P472S F | 5’ GATCAGTTAACGTCCAGCAGTGCAACCTTCCCAG 3’ |
| hNPAS4 P472S R | 5’ GCACTGCTGGACGTTAACTGATCAGAGAAGGTCGCAGTG 3’ |
| hNPAS4 Q500K F | 5' CCTCGGTCAGATCTTATGAAGACAAGTTGACTCCCTGCACCTCC 3' |
| hNPAS4 Q500K R | 5' GGAGGTGCAGGGAGTCAACTTGTCTTCATAAGATCTGACCGAGG 3' |
| hNPAS4 T587M F | 5’ GACTGCATGCTGCTAGCCCTAGCCCAGCTCCG 3’ |
| hNPAS4 T587M R | 5’ TAGGGCTAGCAGCATGCAGTCCCCATTACCAGGGC 3’ |
| hNPAS4 N702D F | 5’ CCTGATGACATGTTTCTAGAAGAGACGCCCGTGGAAG 3’ |
| hNPAS4 N702D R | 5’ CTCTTCTAGAAACATGTCATCAGGGTCAGCCAGGAAGT 3’ |
| hNPAS4 D750Y F | 5’ CTGTCCCCCGAGTACCACAGCTTCCTGGAGGACC 3’ |
| hNPAS4 D750Y R | 5’ GCTGTGGTACTCGGGGGACAGGTTGTTGCAAGGC 3’ |
| hNPAS4 T777I F | 5' CCCCTATGATGGGTTTATTGATGAGCTCCATCAACTCCAGAGC 3' |
| hNPAS4 T777I R | 5' GCTCTGGAGTTGATGGAGCTCATCAATAAACCCATCATAGGG 3' |
| hNPAS4 AscI F | 5' CGATCGCCATGTACCGCTC 3' |
| hNPAS4 F1 | 5' GCTCGTTTAGTGAACCGTCAG 3' |
| hNPAS4 R1 | 5' GCCTTGCAGTGGGCTAGTTAG 3' |
| hNPAS4 F2 | 5' CCCATTTGCCCACCCCATCC 3' |
| hNPAS4 R2 | 5' CTAGTCCGCCAGTGCCAGC 3' |
| hNPAS4 F3 | 5' GAGGCCTCTCCAGTCAAGCAG 3' |
| hNPAS4 R3 | 5' AGGGGTCACAGGGATGCCACCCG 3' |
| hNPAS4 bglI F | 5' GAGGAGATCTGCCGCCGC 3' |
| EF Pst I F | 5’ TTTTTTTCTTCCATTTCAGGTGTCGTGA 3’ |
| SIM1 PstI R | 5’ GAAGGCTGGTTTGGAGGCTG 3’ |
| hSIM1 G254E F | 5' TCCAGGGTGGCGGAGCTCACGGAGTACGAACCTCAGGACCTGA 3' |
| hSIM1 G254E R | 5' TCAGGTCCTGAGGTTCGTACTCCGTGAGCTCCGCCACCCTGGA 3' |
| hSIM1 G254R F | 5' TCCAGGGTGGCGGAGCTCACGCGTTACGAACCTCAGGACCTGA 3' |
| hSIM1 G254R R | 5' TCAGGTCCTGAGGTTCGTAACGCGTGAGCTCCGCCACCCTGGA 3' |
| hSIM1 F160A F | 5' CCCTACCACTCTCACTTCGTGCAGGAGTATGAGATCGAGCGCTCCGCCTTCCTACGTATGAAGTGCGTCTTGG 3' |
| hSIM1 F160A R | 5' ACCTTGTAGCCGCCACAGGTGAGGCCGGCGTTACGCTTGGCCAAGACGCACTTCATACGTAGGAAGGCGGAGC 3' |
| hSIM2L PvuII F | 5' TGCCAAGCTGCTCCCGCTG 3' |
| hSIM2L PvuII R | 5' GGGCCAGCACGCGGTGG 3' |
| hSIM2L F160A F | 5' GAGTATGAGATAGAGAGATCTGCCTTTCTTCGAATGAAATGTGTCTTGG 3' |
| hSIM2L F160A R | 5' ATTTCATTCGAAGAAAGGCAGATCTCTCTATCTCATACTCTTGGAGCAGG 3' |
